# 

**Published:** 1882-11

**Authors:** 


### CONTENTS:

PAGE.

ORIGINAL COMMUNICATIONS:

I. Traumatic Tetanus. Recovery under Quinine and Alcohol. Sphæro Bacteria Present in the Blood. By Daniel R. Brower, M.D ........ 450

II. Micro Organisms Found in the Blood of a case of Tetanus. By Lester Curtis, M.D........ 462

III. A Simple Suction Drainage Tube for Suppurating Pleural Cavities. By Edmund Andrews, M.D........ 469

IV. Agoraphobia. A Contribution to Clinical Medicine. By L. T. Potter, B L., M.D........ 472

V. The Actual Cautery in Cancer of the Uterus. By O. Stroinski, M.D........ 476

VI. Chronic Cystitis in the Daughter; Fibroid Polypus of the Bladder in the Mother; Extirpation and Laceration of the Bladder; Suture; Recovery. By O. Stroinsky, M.D...... 478

SOCIETY REPORTS:

VII. American Dermatological Association. Proceedings at the Sixth Annual Meeting........ 481

PAGE.

VIII. Local Societies.

XI. Chicago Pathological Society........ 516

X. Chicago Biological Society........ 518

DOMESTIC CORRESPONDENCE:

XI. New York Letters........ 522, 529

XII. Letter from Tama City, Iowa........ 531

XIII. Letter from Elgin........ 532

EDITORIAL:

The Latest Sphæro Bacterium—A Good Thing to Have in the Family........ 534

TRANSLATIONS FROM FOREIGN EXCHANGES...... 539

SELECTIONS:

A Case of Severe Injury of the Orbit. By E. L. Holmes, M.D........ 555

OBITUARY........ 559

ITEMS........521, 533, 538

ANNOUNCEMENTS FOR THE MONTH........ 560

NOTICE TO CORRESPONDENTS........*advts.*

